# P-835. The microbiological characteristics of *Acinetobacter baumannii* associated with early mortality in patients with bloodstream infection

**DOI:** 10.1093/ofid/ofae631.1027

**Published:** 2025-01-29

**Authors:** Chan Mi Lee, Yunsang Choi, Seong Jin Choi, Song Mi Moon, Eu Suk Kim, Hong Bin Kim, Sin Young Ham, Jeong Su Park, Jinki Yeom, Kyoung-Ho Song

**Affiliations:** Seoul National University College of Medicine, Seoul, Seoul-t'ukpyolsi, Republic of Korea; Seoul National University Bundang Hospital, Seoungnam-si, Kyonggi-do, Republic of Korea; Seoul National University Bundang Hospital, Seoungnam-si, Kyonggi-do, Republic of Korea; Seoul National University College of Medicine, Seoul, Seoul-t'ukpyolsi, Republic of Korea; Seoul National University Bundang Hospital, Seoungnam-si, Kyonggi-do, Republic of Korea; Seoul National University College of Medicine, Seoul, Seoul-t'ukpyolsi, Republic of Korea; Seoul National University Bundang Hospital, Seoungnam-si, Kyonggi-do, Republic of Korea; Department of Laboratory Medicine, Seoul National University Bundang Hospital, Seoul, Seoul-t'ukpyolsi, Republic of Korea; Seoul National University College of Medicine, Seoul, Seoul-t'ukpyolsi, Republic of Korea; Seoul National University College of Medicine, Seoul, Seoul-t'ukpyolsi, Republic of Korea

## Abstract

**Background:**

Despite the rapid deaths due to *Acinetobacter baumannii* bacteremia, the clinical impact of the microbiological characteristics of *A. baumannii* strains on early mortality (EM) is unclear. We aimed to identify the microbiological characteristics of *A. baumannii* strains associated with EM.

**Figure:**
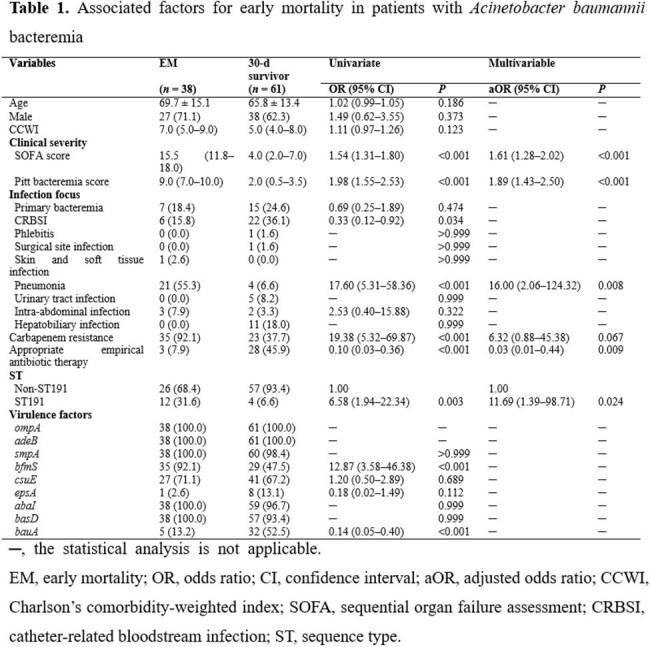

**Methods:**

Clinical information and isolates from patients with *A. baumannii* bacteremia from January 2015 to December 2021 were collected. We collected patients’ clinical data, including demographic data, clinical severity, source of infection, and antibiotic therapy. EM was defined as death within 3 d of the initial positive blood culture, whereas late mortality meant death within 5–30 d. The microbiological characteristics of *A. baumannii* were analyzed using multilocus sequence typing, polymerase chain reactions, and a *Galleria mellonella in vivo* infection model.

**Figure 1.**
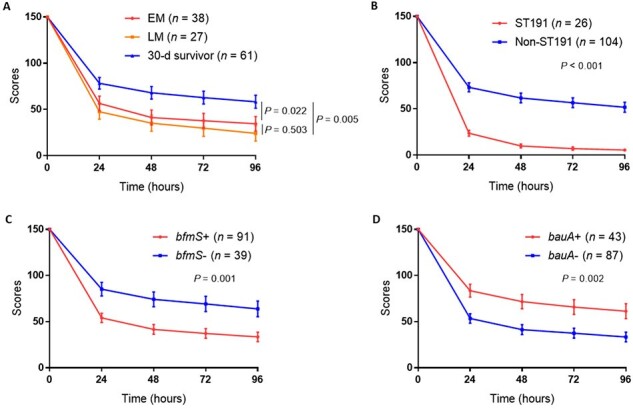
The virulence of Acinetobacter baumannii isolates by Galleria mellonella infection model. (A) Health index scores of larvae injected with isolates from the early mortality (EM), late mortality (LM), and 30-d survivor groups. (B) Health index scores of larvae injected with ST191 and non-ST191. (C) Health index scores of larvae injected with isolates carrying bfmS and those without bfmS. (D) Health index scores of larvae injected with isolates carrying bauA and those without bauA. The circles, squares, and triangles represent the mean, and the lines indicates standard error of the mean. The health index score of larvae was calculated by summing the scores for activity, cocoon formation, melanization, and survival. Health index scores of fifteen larvae were summed at every 24-h time point.

**Results:**

Among 130 patients, 69 (53.1%) died within 30 d, and 38 (55.1% of 30-d deaths) were classified as EM and 27 (39.1% of 30-d deaths) as LM. Sequence type 191 (ST191) was the most prevalent ST, consistently identified throughout the study period. ST191 strain was more prevalent patients with EM than in 30-d survivors (31.6% *vs*. 6.6%, *P* = 0.001). Regarding virulence genes, *bfmS* was more frequent (92.1% *vs*. 47.5%, *P* < 0.001), whereas *bauA* was less frequent (13.2% *vs*. 52.5%, *P* < 0.001) in patients with EM than in 30-d survivors. Higher clinical severity, pneumonia, and ST191 infection were identified as independent risk factors for EM. In the *G. mellonella* infection model, ST191, *bfmS*+, and *bauA*- isolates showed higher virulence than non-ST191, *bfmS*-, and *bauA*+ isolates, respectively.

**Conclusion:**

ST191 and *bfmS* were more frequently found in the EM group. ST191 infection was also an independent risk factor for EM and highly virulent in the *in vivo* model. Tailored infection control measures based on these characteristics are necessary for *A. baumannii* bacteremia management.

**Disclosures:**

**All Authors**: No reported disclosures

